# MYC selects against reduced BCL2A1/A1 protein expression during B cell lymphomagenesis

**DOI:** 10.1038/onc.2016.362

**Published:** 2016-10-03

**Authors:** M Sochalska, F Schuler, J G Weiss, M Prchal-Murphy, V Sexl, A Villunger

**Affiliations:** 1 Division of Developmental Immunology, Biocenter, Medical University of Innsbruck, Innsbruck, Austria; 2 Institute of Pharmacology and Toxicology, Department of Biomedical Sciences, University of Veterinary Medicine, Vienna, Austria; 3 Tyrolean Cancer Research Institute, Innsbruck, Austria

## Abstract

Rearrangements of MYC or ABL proto-oncogenes lead to deregulated expression of key-regulators of cell cycle and cell survival, thereby constituting important drivers of blood cancer. Members of the BCL-2 family of apoptosis regulators contribute to oncogenic transformation downstream of these oncogenes, but the role of anti-apoptotic BCL2A1/A1 in transformation and drug resistance caused by deregulation of these oncogenes remains enigmatic. Here we analyzed the role of A1 in MYC as well as ABL kinase-driven blood cancer in mice, employing *in vivo* RNAi. We report that overexpression of either oncogene leads to a significant increase in A1 protein levels in otherwise A1-negative B cell progenitors, indicating a key role downstream of these oncogenes to secure survival during transformation. Knockdown of A1 by RNAi, however, did not impact on tumor latency in v-Abl-driven pre-B-ALL. In contrast, A1 knockdown in premalignant *Eμ-MYC* mice caused a significant reduction of transgenic pre-B cells without impacting on tumor latency as the emerging lymphomas escaped silencing of A1 expression. These findings identify A1 as a MYC target that can be induced prematurely during B cell development to aid expansion of otherwise cell-death-prone MYC transgenic pre-B cells. Hence, A1 should be considered as a putative drug target in MYC-driven blood cancer.

## Introduction

The role of anti-apoptotic BCL-2 family members as disease promoters and mediators of drug resistance in human cancer is well established. This prompted the development of BCL2 inhibitors, some of them well advanced in clinical trials, with one of them recently approved for the treatment of refractory chronic lymphocytic leukemia (CLL).^1^

Despite the large degree of redundancy of individual BCL-2 family proteins upon overexpression, cell type and trigger-specific survival dependences have been noted. This led to the concept of ‘BCL-2-family addiction' of human cancers, which means that tumor cells depend regularly on one particular BCL-2 family protein for cell survival, despite the fact that more such proteins are found expressed in a given cancer cell type.^2^ Employing a rapid screening method, referred to as ‘BH3-profiling', the dependence of a cancer cell on a subset of BCL-2 prosurvival family members (BCL-2, BCL-X, BCL-W, MCL-1 or A1/BFL-1) can be predicted with high reliability, facilitating choice of treatment.^3^ Recent studies in human cancer cells and cell lines, as well as different animal models of blood cancer, including those for BCR-ABL-driven pre-B-ALL or MYC-driven B cell lymphomas, have assigned critical survival roles to anti-apoptotic MCL-1 with sometimes auxiliary roles for other survival factors, mainly BCL-X, but quite often dispensable roles for BCL-2 itself.^4, 5^

Although the key-role of MCL-1 or BCL-2 in tumor cell survival and drug resistance is undisputed, little is known about the relevance of related BCL2A1/A1 (called BFL-1 in humans), a poorly investigated member of the BCL-2 family. A1 has been implicated as tumor promoter or drug-resistance factor in different types of lymphoid malignancies, including pre-B acute lymphoblastic leukemia (pre-B-ALL), B chronic lymphocytic leukemia (B-CLL), mantle lymphoma (ML) or diffuse large-B cell lymphoma (DLBCL) (reviewed in Ottina *et al.*^6^; Vogler *et al.*^7^ CLL and DLBCL patients with high *BFL1* mRNA expression levels show poor prognosis and increased resistance to chemotherapeutics.^8^ More recent studies further describe BFL-1 also as a resistance factor in BRAF-targeting therapy in melanoma and BCL-2 inhibitor-treated CLL.^9, 10, 11^ A1/BFL-1 is thus considered a putative therapeutic target in human cancer, warranting its exploration in preclinical models.

Although rats and humans contain one gene encoding for A1/BFL-1, mice contain four genes, three of them encoding for the functional paralogues *A1a*, *A1b* and *A1d*. These three share more than 95% homology at the protein as well as at the DNA level, whereas *A1c* encodes a pseudogene.^12^ Because of this complex genetic organization of the *Bcl2a1* locus in mice, no functional studies have been performed, leaving the role of A1 in preclinical models of cancer unexplored. In normal tissues of adult mice, A1 is expressed at low level in the hematopoietic system, in both lymphoid and myeloid cells, but rapidly induced upon antigen-receptor stimulation in T and B cells or inflammatory cytokines as well as lipopolysaccharide in myeloid cells and FcɛRI ligation in mast cells.^13, 14, 15^ Further evidence for a role of A1 in lymphocyte survival originates from experiments where expression was reduced by RNAi *in vivo*. Here B cells show increased spontaneous and BCR-induced apoptosis in culture and, depending on the RNAi system used, fail to accumulate in normal numbers.^14, 16^ This prompted us to test whether A1 is a putative therapeutic target in B cell malignancies and whether B lymphoid tumors may be addicted to A1 expression for survival. Therefore, we employed an RNAi-based constitutive A1 knockdown mouse model to elicit its effects on tumorigenesis induced by two common oncogenic drivers of hematological disorders, c-MYC and ABL kinase. Our findings provide evidence for a rate-limiting role of A1 in MYC-driven B cell lymphomagenesis.

## Results

### Abelson kinase-driven oncogenic stress increases A1 protein expression

**Figure 1:**
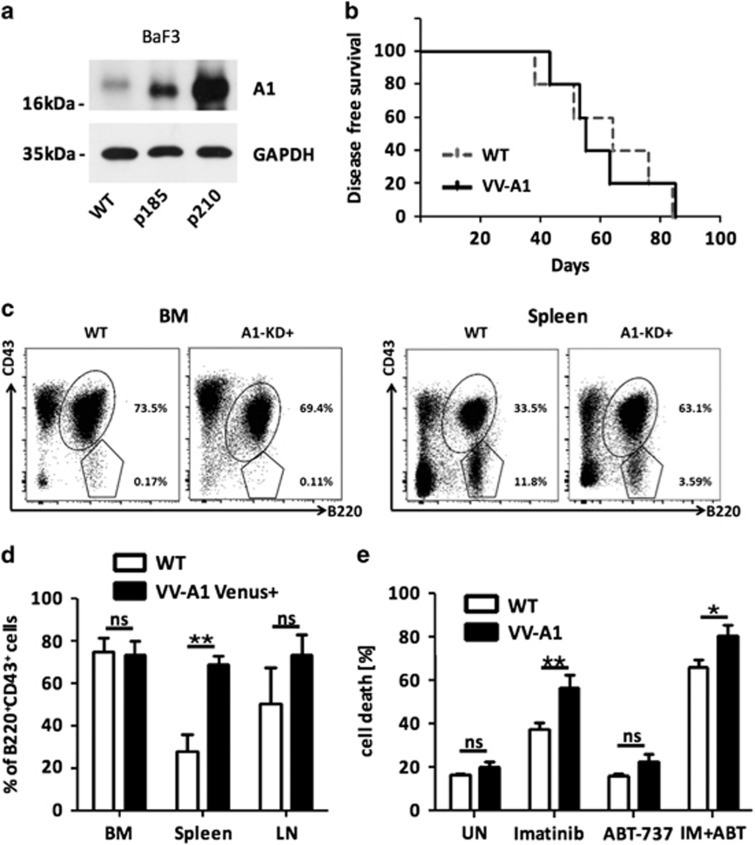
A1 expression is induced upon ABL kinase-driven transformation and contributes to drug sensitivity. (**a**) A1 expression was assessed in parental BaF3 mouse pro-B cells and in cells stably transduced with p185^BCR-ABL^ or p210^BCR-ABL^ oncogenes. (**b**) Kaplan–Meier analysis of pre-B-ALL progression in v-Abl-infected WT and VV-A1 mice (*n*=5); *P*= 0.9399 (Mantel–Cox). (**c**) Representative dot plots of flow cytometric analysis of diseased organs showing accumulation of leukemic CD43^+^B220^+^ immature and CD43^−^B220^+^ mature B cells, quantified in (**d**). Percentages of cells falling within each gate are shown. ANOVA followed by Bonferroni *post hoc* test. **P⩽0.01. (*n*=5, biological replicates, five independent experiments). (**e**) Data are derived from five different cell lines established from diseased wild type or VV-A1 v-Abl-infected mice that were treated for 24 h with 2 μM imatinib, 1 μM ABT-737 or both. Viability was assessed by Annexin V staining and flow cytometry after 24 h. Bars represent means±s.e.m. (*n*=5, biological replicates, three independent experiments). Data were analyzed by ANOVA followed by Bonferroni *post hoc* test. *P⩽0.05; **P⩽0.01. BM=bone marrow, LN=lymph nodes, IM=imatinib.

Many members of the BCL-2 family are deregulated upon oncogenic transformation in mouse or human cells. To test whether A1 is also affected under such conditions, we analyzed the impact of aberrant ABL-kinase signaling on A1 protein expression. Similar to total bone marrow or early B cells in mice,^14^ IL3-dependent BaF3 pro-B cells do express only low amounts of A1 protein. However, upon transformation with the p185 or the p210 variant of the BCR-ABL oncogene, protein expression was strongly elevated (Figure 1a), a fact that correlated well with their gained growth factor independence (not shown). Together, this suggests that oncogenic ABL-kinase signaling increases A1 protein levels, possibly to implement factor-independent cell survival, and that A1 may be a relevant player for tumorigenesis or drug responsiveness in BCR-ABL^+^ pre-B-ALL.

### v-Abl-driven pre-B-ALL is not limited by A1 knockdown

To investigate the relevance of A1 in ABL kinase-driven malignancies, we took advantage of our recently established mouse model. In these mice, all A1 paralogues expressed are constitutively targeted by a single shRNA embedded in the miR30 backbone, placed in the 3′UTR of the fluorescence marker Venus and expressed under control of the hematopoiesis specific *Vav*-gene promoter (VV-A1 mice).^16^ Depending on the organ, more than 60% of all hematopoietic cells express the Venus reporter, indicative of significant, albeit mosaic, transgene expression (Supplementary Figure S1a). Venus reporter expression was shown by us to correlate well with *A1* mRNA knockdown, ranging from more than 80% in Venus^+^ thymocytes or bone marrow cells to about 60% in the spleen.^16^ Of note, this mouse strain shows no gross abnormalities in leukocyte subset composition, neither in the Venus^+^ nor Venus-negative pool of cells, when compared with wild-type (WT) or transgenic mice expressing a control shRNA targeting Firefly luciferase (VV-FF mice) (Supplementary Figure S1b and Ottina *et al.*^16^

To address the role of A1 in leukemia development, we first performed colony-formation assays using primary bone marrow from these animals that was transduced with p185^BCR-ABL^.^17^ No appreciable differences in colony formation were observed between genotypes (data not shown). Next, we infected newborn WT or VV-A1 mice with p160^v-Abl^ encoding retrovirus and monitored leukemia latency (Figure 1b). Infected mice of both genotypes developed immature B cell precursor (pre-B) ALL with similar penetrance, and latency and organ analysis by flow cytometry confirmed comparable disease burden in bone marrow, spleen and lymph nodes (Figures 1c and d; not shown). Surprisingly, spleens from VV-A1 mice showed even higher percentages of B220^+^CD43^+^ tumor cells when compared with WT controls, whereas B220^+^CD43^−^ B cells, representing more mature cells, were actually lost (Figures 1c and d). It is hence tempting to speculate that mature B cells suffer a survival disadvantage upon A1 knockdown under conditions of stress,^14^ which may render them less competitive in a tumor-infiltrated environment.

We next established v-Abl immortalized cell lines derived from the bone marrow of diseased mice. These cell lines were tested for their ability to respond to therapy. Cells were treated with imatinib (Gleevec, Selleckchem, Munich, Germany), a clinically used BCR-ABL inhibitor that induces apoptosis depending on the BH3-only protein and established A1 antagonist, BCL-2 interacting mediator of cell death (BIM).^18^ In addition, we tested the response of these tumor cells to the BH3-mimetic ABT-737 that inhibits BCL-2, BCL-X and BCL-W but not MCL-1 or A1.^19^ Compared with immortalized WT controls, cells derived from VV-A1 knockdown mice were found more responsive to imatinib or imatinib plus ABT-737 (Figure 1e).

These data suggest that the ABL kinase-driven increase in A1 expression is not essential for tumor development, indicating functional redundancy with other survival proteins. Alternatively, the knockdown efficacy achieved in pre-B cells may not suffice to reveal a rate-limiting effect. Intriguingly, reduction of A1 expression sensitizes Abl-dependent tumor cells to imatinib, thereby opening a potential therapeutic avenue to improve treatment efficacy of ABL-kinase inhibitors in BCR-ABL-driven disease.

### MYC deregulation uncouples A1 expression from BCR signaling

**Figure 2:**
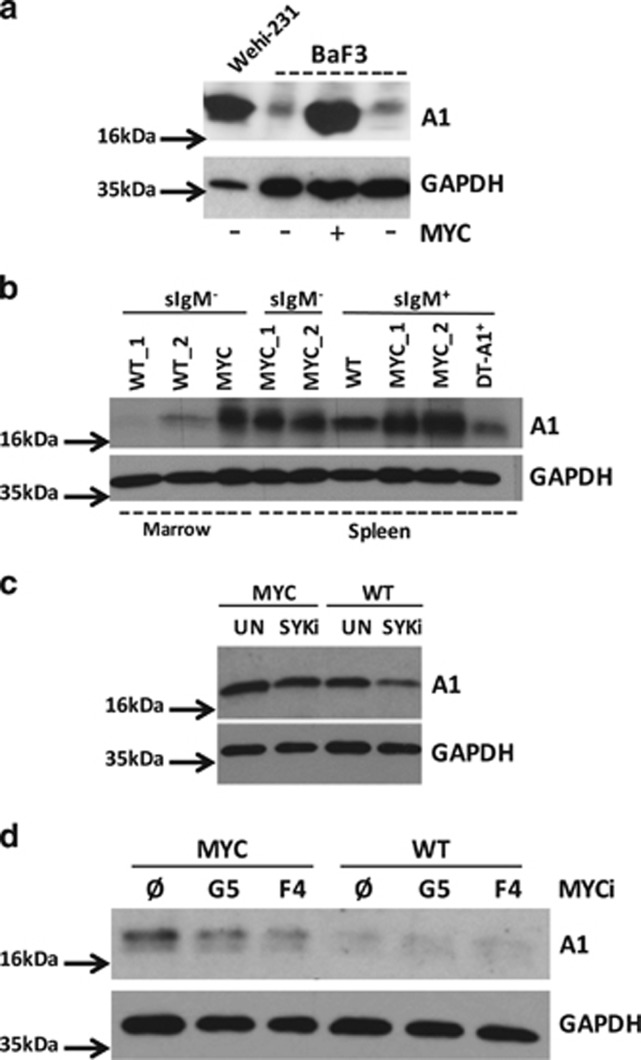
Deregulated MYC expression increases A1 protein levels. A1 protein level was assessed in (**a**) parental BaF3 mouse pro-B cells and in cells stably transformed with c-MYC or empty vector (EV). (**b**) Sorted bone marrow or splenic B220^+^ sIgM^+^ or sIgM^−^ B cells derived from wild-type and premalignant *Eμ-MYC* or *DT-A1* transgenic mice 4–6 weeks of age were assessed for A1 expression by immunoblot. Figure displays biological replicates; one out of two independent experiments is shown. (**c**) Expression of A1 in sorted B220^+^ splenocytes from *Eμ-MYC* or wild-type mice cultured for 4 h in the absence or presence of 5 μM R406 (SYK inhibitor). One out of two experiments is shown. (**d**) Sorted IgM^+^B220^+^ splenocytes from *Eμ-MYC* or wild-type mice were cultured for 16 h in the absence or presence of MYC inhibitors 10074-G5 (G5) or 10058-F4 (F4), 25 μM each. Membranes were reprobed for GAPDH to control for protein loading. Figure displays one out of two independent experiments.

To test if also the MYC oncogene influences A1 expression in B cells, we analyzed A1 protein levels in BaF3 cells transduced with a c-MYC encoding retrovirus or a control (Figure 2a). Along the same line, we analyzed A1 expression in sorted bone marrow or splenic B220^+^ sIgM^+^ and sIgM^−^ B cells from WT or premalignant *Eμ-MYC* or *DT-A1* transgenic mice (Figure 2b). Here both BaF3 c-MYC cells and *Eμ-MYC* transgenic sIgM-negative B cells showed substantially higher A1 levels than their respective controls (Figures 2a and b). sIgM-negative B cells isolated form *Eμ-MYC* transgenic spleens showed a similar increase in A1 expression than those isolated from bone marrow. In addition, sIgM^+^ splenic B cells, known to express basal levels of A1,^14^ increase A1 levels further in the presence of oncogenic levels of MYC. Of note, A1 expression was reduced by about half in Venus^+^ cells derived from *Eμ-MYC x VV-A1* double-transgenic (DT-A1) mice (Figure 2b). The latter finding is in line with the previously reported degree of *A1* mRNA repression in Venus^+^ splenocytes of VV-A1 mice.^16^

Since we have shown that A1 levels in follicular B cells are regulated by BCR signaling,^14^ we reasoned that A1 expression in the context of MYC overexpression might become uncoupled from these signals. To test this concept, we first investigated whether SYK inhibition is still capable to reduce A1 expression in MYC transgenic B cells. Inhibition of SYK in WT cells that lack exogenous MYC expression reduced A1 levels, but no effect on A1 expression could be observed in MYC-transgenic IgM^+^ B cells (Figure 2c). In line with our hypothesis, the treatment of sIgM^+^ MYC transgenic B cells with two different MYC inhibitors (G5 or F4) led to an appreciable reduction of A1 protein expression (Figure 2d), supporting the idea that A1 is placed under control of c-MYC in this context.

### MYC selects for sustained A1 expression during transformation

**Figure 3:**
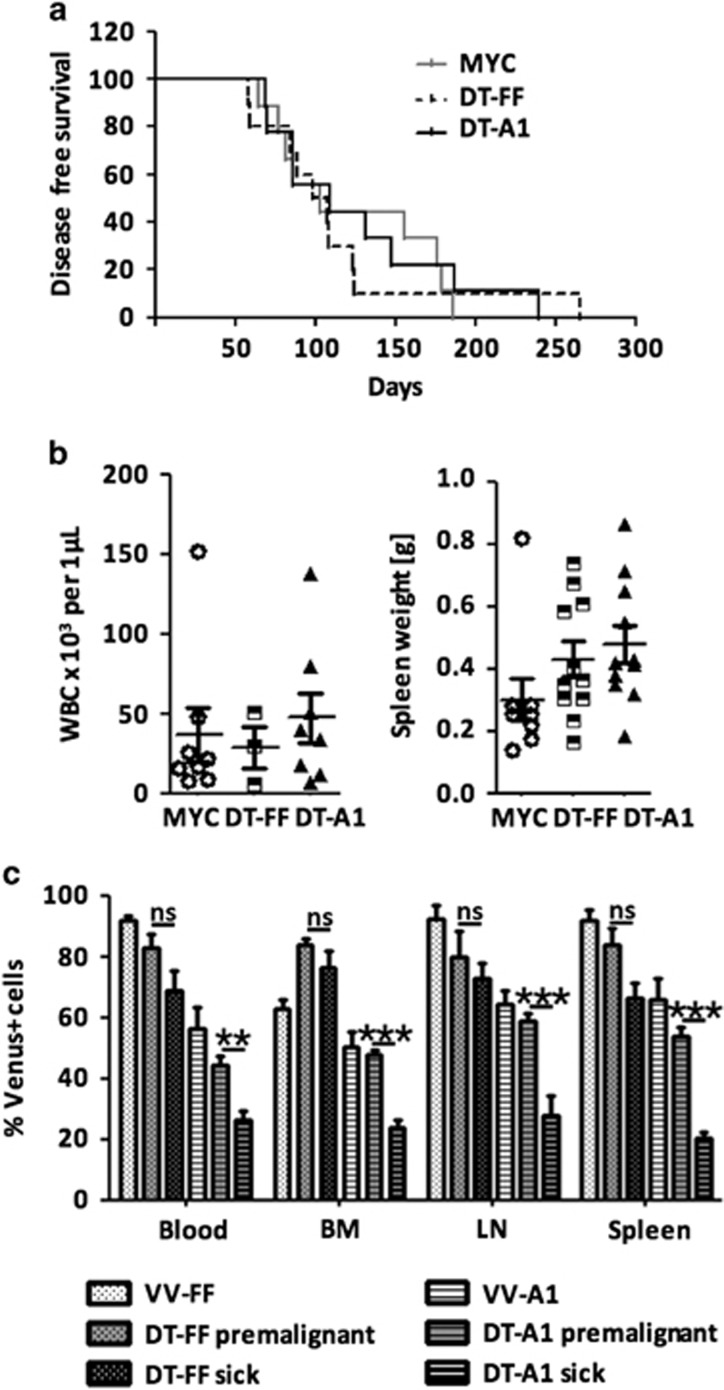
A1 knockdown cells are counter-selected in *Eμ-MYC*-driven lymphomas. (**a**) Kaplan–Meier analysis of disease-free survival of mice of the indicated genotypes (MYC *n*=8, DT-FF *n*=10, DT-A1 *n*=11). MYC vs DT-A1 *P*=0.6093; DT-FF vs DTA1 *P*=0.6345 (Mantel–Cox); median survival: MYC 103 days, DT-FF 102.5 days, DT-A1 109 days. (**b**) Left panel: white blood cell counts in the blood of moribund mice. Dots represent individual blood count in diseased mice and bars represent the corresponding means (MYC *n*=8, DT-FF *n*=3, DT-A1 *n*=8 mice). Right panel: Spleen weights of diseased mice of the indicated genotypes (MYC *n*=7, DT-FF *n*=10, DT-A1 *n*=11 mice). No significant differences were noted. (**c**) Quantification of the fraction of Venus^+^ cells in primary and secondary lymphatic organs derived from premalignant and diseased DT-FF and DT-A1 mice (VV-FF *n*=3, VV-A1 *n*=5, DT-FF premalignant *n*=5, sick *n*=5; DT-A1 premalignant *n*=11, sick *n*=6; biological replicates, six independent experiments). Bars represent means±s.e.m. ANOVA followed by Bonferroni *post hoc* test. **P⩽0.01, ***P⩽0.001.

On the basis of these observations, we decided to study the role of A1 in MYC-driven lymphomagenesis by intercrossing *VV-A1* or *VV-FF* mice, expressing a miR-shRNA-targeting firefly luciferase, with *Eμ-MYC* transgenic mice. *Eμ-MYC* transgenic mice develop aggressive pre-B sIgM^−^ or sIgM^+^ B cell lymphomas within the first year of life.^20^ *Eμ-MYC* mice harboring A1 knockdown (referred to as DT-A1 mice) did not show differences in disease-free survival when compared with single transgenic *Eμ-MYC* or *Eμ-MYC* mice expressing the control miR-shRNA (DT-FF mice). Accordingly, disease burden, as assessed by white blood cell counts, and splenic weight were comparable across genotypes (Figures 3a and b).

Loss of essential survival factors, such as Mcl-1, often selects for tumors that have escaped Cre-mediated deletion.^21, 22^ We thus compared transgene expression, as indicated by the Venus reporter, in 4–6-weeks-old premalignant mice with reporter expression in diseased mice (Figure 3c). Flow cytometric analysis revealed that the percentage of Venus-expressing cells was significantly reduced in bone marrow, blood, lymph node and spleen of leukemic DT-A1 mice, suggesting possible counter-selection of cells expressing the miR-shRNA targeting A1. Importantly, we failed to detect reduced Venus expression in diseased DT-FF double transgenic mice (Figure 3c). These data led us to conclude that high MYC expression selects against miR-shRNA expression in DT-A1 mice in an attempt to sustain A1 levels.

### MYC-transgenic B cells with reduced A1 levels are more susceptible to apoptosis

**Figure 4:**
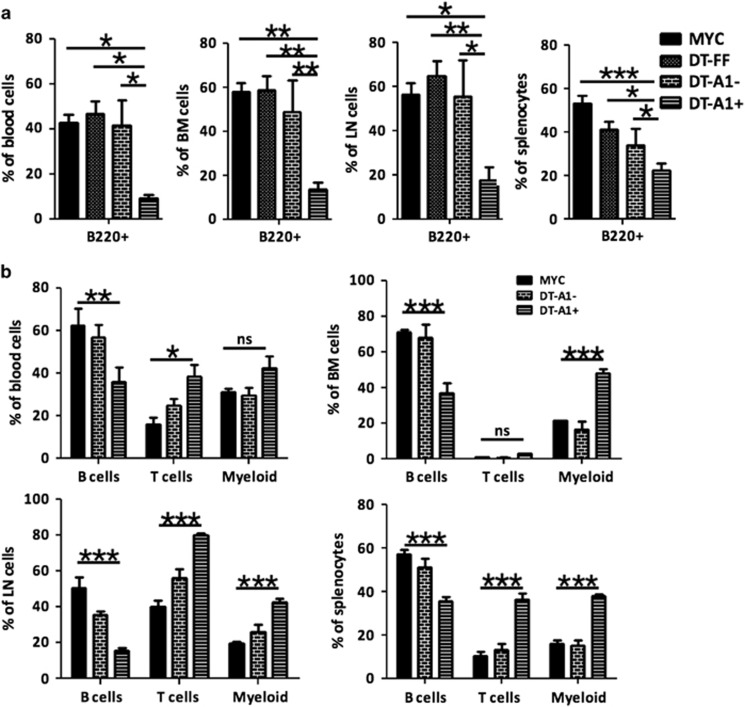
A1 knockdown leads to the loss of leukemic B cells. (**a**) Quantification of B cells in the spleen, bone marrow, blood and axillary lymph nodes of diseased mice. Single cell suspensions were stained with anti-B220 antibodies. Individual bars depict the percentage of B220^+^ B cells from the total cell fraction of MYC or DT-FF mice (>90% Venus^+^ cells) as well as of Venus^−^ (DT-A1^−^) and Venus^+^ (DT-A1^+^) cells from DT-A1 mice. Bars represent means±s.e.m. (MYC *n*=4, DT-FF *n*=5, DT-A1 *n*=6, biological replicates). ANOVA followed by Bonferroni *post hoc* test. *P⩽0.05, **P⩽0.01, ***P⩽0.001. (**b**) Analysis of leucocyte subset distribution in Venus^+^ (DT-A1^+^) and Venus^−^ (DT-A1^−^) cell fractions of premalignant 4–6-week-old DT-A1 mice in comparison to *Eμ-MYC* control mice. LN=lymph nodes, BM=bone marrow, PB=peripheral blood. Bars represent means±s.e.m. (MYC *n*=4, DT-FF *n*=5, DT-A1 *n*=6 biological replicates, three independent experiments). ANOVA followed by Bonferroni *post hoc* test. *P⩽0.05, **P⩽0.01, ***P⩽0.001.

To test whether the loss of Venus expression, as noted in leukemic mice, was indeed due to loss of transgene expression in B cells, we performed more detailed flow cytometric analyses on isolated tumors. Consistent with our hypothesis of counter-selection, the majority of B220^+^ B cells were found to be Venus-negative in DT-A1 mice, whereas DT-FF tumors remained Venus^+^ in blood, spleen, bone marrow or lymph nodes of moribund mice (Figure 4a; Supplementary Figure S2).

To dissect at which stage of B cell development cells expressing the miR-shRNA targeting A1 were counter-selected, early during development or upon transformation, we analyzed the Venus expression levels in premalignant mice at 5–6 weeks of age. Flow cytometric analysis revealed that Venus-positive pre-B cells were already significantly underrepresented in bone marrow, spleen, blood or lymph nodes of premalignant mice. This phenomenon was accompanied by the proportional increase in T and myeloid cells (Figure 4b; Supplementary Figure S2).

**Figure 5:**
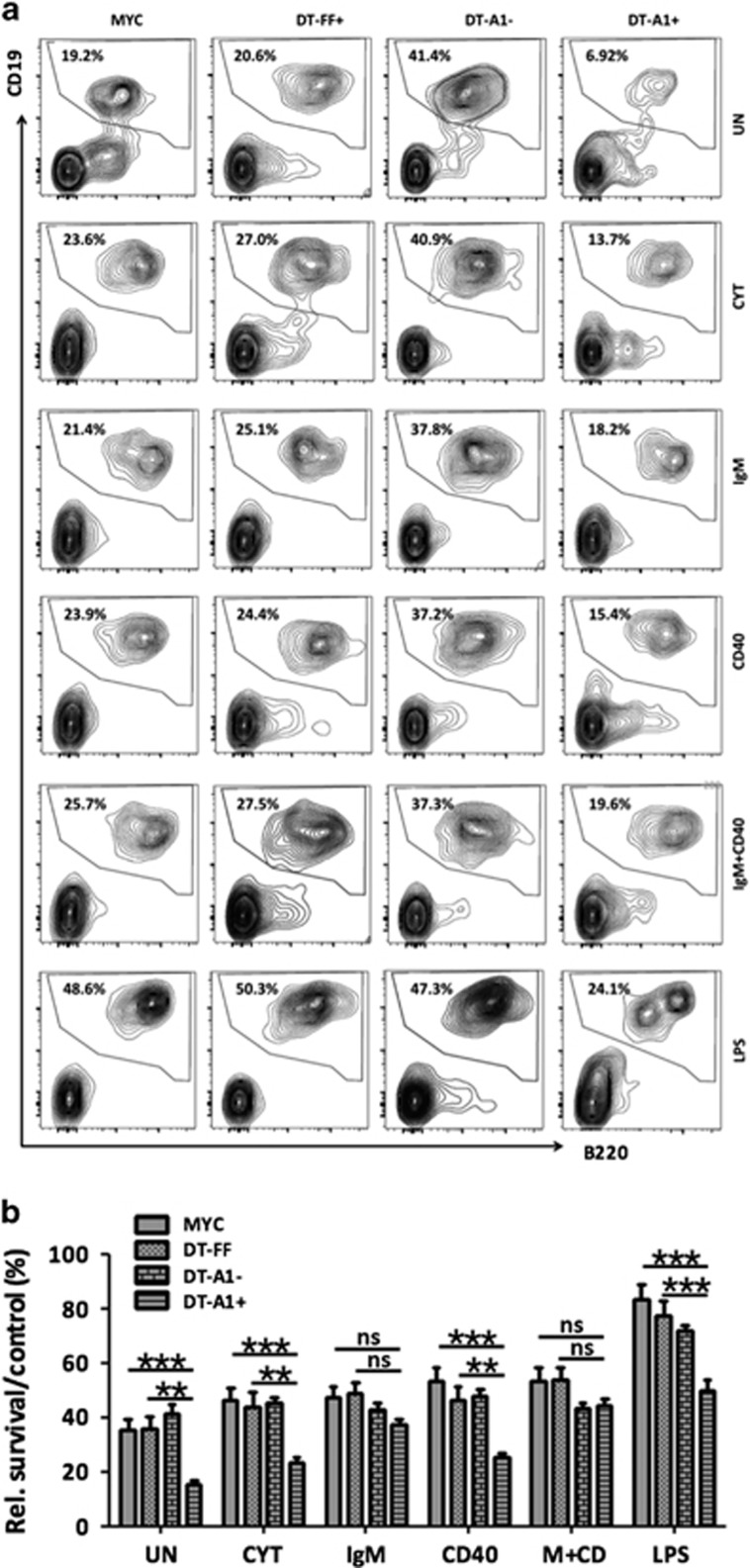
Impaired survival of premalignant splenic B cells upon A1 knockdown. Total spleen cells from mice of the indicated genotypes were incubated in the presence or absence of the indicated mitogens for 24 h. The percentages of viable (Annexin V^−^) CD19^+^B220^+^ B cells in the Venus^−^ or Venus^+^ fractions derived from DT-A1 mice or the total cell fraction of MYC or DT-FF mice (>90% Venus^+^ cells) was assessed by flow cytometry. Representative contour plots of mitogen-treated splenocyte cultures stained with anti-CD19 and anti-B220-specific antibodies and percentages of cells falling within each gate are shown. (**b**) Quantification of data shown in (**a**). To assess relative survival, the percentage of viable B cells was compared with the percentage of B cells identified straight after sacrifice. As these percentages differ, the bar graphs show the ratio of the percentage of B220^+^CD19^+^ B cells in culture after 24 h, divided by the percentage of the B220^+^ B cells present in the spleen on the day of sacrifice. All experiments were performed in technical duplicates. Bars represent means±s.e.m. (MYC *n*=8, DT-FF *n*=5, DT-A1 *n*=13, biological replicates, six independent experiments). ANOVA followed by Bonferroni *post hoc* test. **P⩽0.01, ***P⩽0.001. UN=untreated, CYT=cytokines (IL-2, IL-4 and IL-5), M+CD=anti-IgM and anti-CD40.

This suggested that the reduction of A1 expression in *DT-A1* mice affects survival of premalignant B cells. Therefore, splenocytes from *Eμ-MYC*, *DT-FF* and *DT-A1* were put in culture, either left untreated or stimulated with mitogens known to induce A1 expression. After 24 h, we analyzed the percentage of viable B cells compared with that detected straight after isolation. In line with a role for A1 in B cell survival in the context of MYC overexpression, we observed a strong reduction in the percentage of viable B cells in *DT-A1* splenocyte cultures, in relation to Venus-expression, when compared with *Eμ-MYC* or *DT-FF* controls. Significant differences were observed in untreated cultures but also upon stimulation with IL2/4/5, lipopolysaccharide or anti-CD40. Although all these treatments increased B cell survival *ex vivo* to a certain degree when compared with untreated controls, IgM stimulation selectively improved the survival of MYC-transgenic B cells expressing the shRNA targeting A1 (DT-A1^+^) (Figures 5a and b). As this effect was not noted in Venus-negative cells or cells derived from the other genotypes, we speculate that the IgM-driven increase in *A1* mRNA transcription and protein expression may exceed the capacity of the A1 knockdown system, thereby facilitating the survival of these cells. We conclude that tight control of A1 expression is needed to prevent cell death upon oncogenic stress caused by deregulation of c-MYC in premalignant B cells.

### BFL-1 transgene expression rescues B cell loss in DT-A1 mice

**Figure 6:**
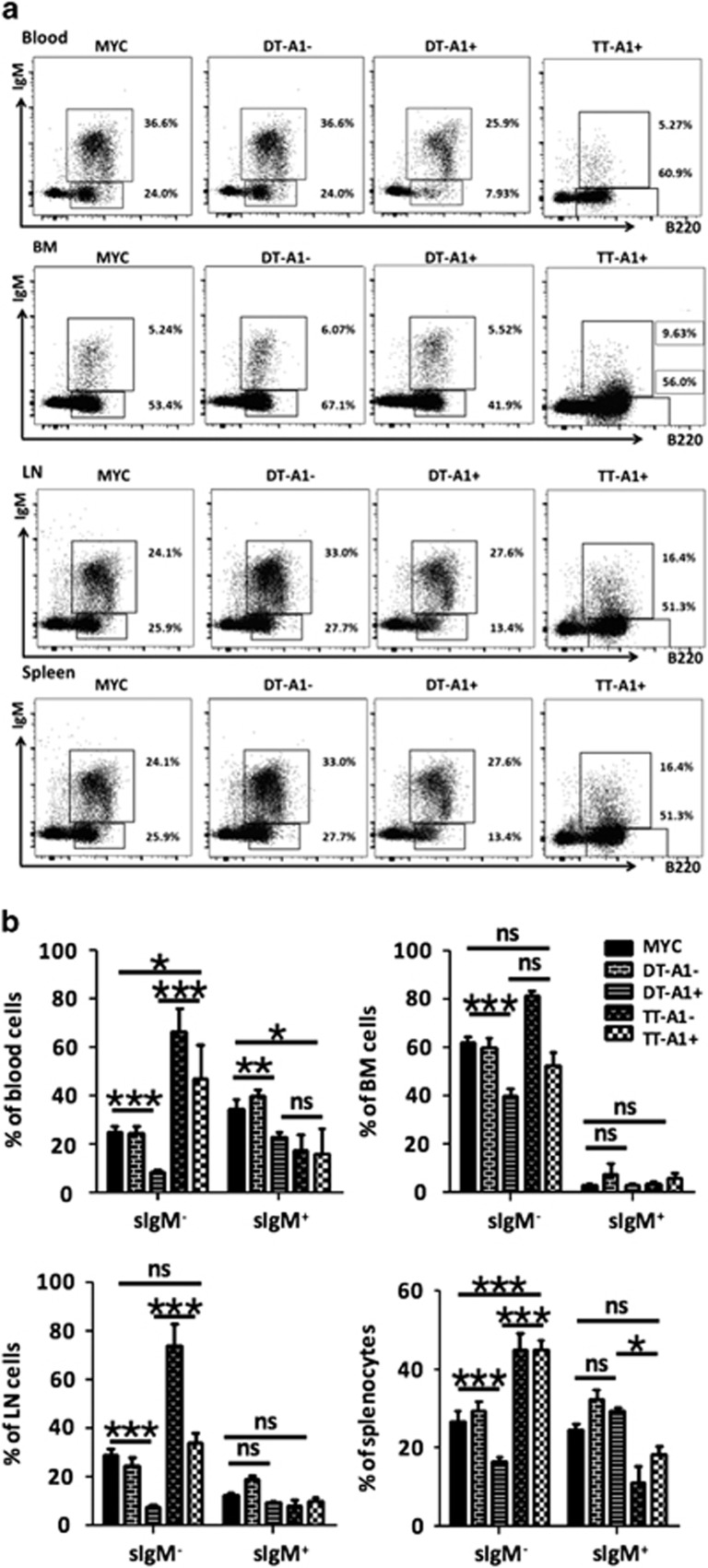
Transgenic overexpression of *BFL-1* rescues premalignant B cells from the effects of A1 knockdown. Flow cytometric analysis of single-cell suspensions from blood, bone marrow, lymph nodes and spleen. (**a**) Representative dot-plots showing percentages of sIgM^+^ and sIgM^−^ B cells in major lymphatic organs. Cells from premalignant mice of the indicated genotypes were stained with antibodies recognizing B220 or IgM, and analyzed further based on Venus-reporter expression (IgM^−^ cells representing pro/pre-B cells and IgM^+^ cells representing immature/mature B cells). The percentage of cells falling within each gate is shown. Data shown in (**a**) are quantified in **(b)**. Bars represent means±s.e.m. (MYC *n*=8, DT-A1 *n*=13, TT-A1 *n*=4 biological replicates, six independent experiments). ANOVA followed by Bonferroni *post hoc* test. *P⩽0.05, **P⩽0.01, ***P⩽0.001. LN=lymph nodes, BM=bone marrow. NS=not significant.

Despite the use of DT-FF mice as a model system controlling for generic side effects caused by miR-shRNA overexpression, it remained possible that the increased cell death susceptibility and underrepresentation of Venus^+^ MYC-transgenic B cells noted in DT-A1 mice might be due to a miR-shRNA-driven off-target effect rather than a reduction in A1 expression. To exclude this possibility, we intercrossed *DT-A1* mice with animals overexpressing human *BFL-1* as a transgene under control of the *Vav*-gene promoter (Supplementary Figure S3a). Although *Vav-BFL-1* transgenic mice did not show a substantial overexpression of BFL-1 protein in the bone marrow when compared with lymph node, thymus or spleen, nor an increase in pro/pre-B cells (Supplementary Figure S3a; Tuzlak *et al.*, manuscript in preparation), transgene expression clearly prevented the loss of sIgM-negative B cells in triple transgenic (TT-A1) mice (Supplementary Figure S3b). In fact, sIgM-negative B cells became the dominant B cell type in all lymphatic organs in these animals (Figures 6a and b). This observation suggested that even low-level BFL-1 overexpression compensates for the shRNA-mediated knockdown of A1 in the presence of oncogenic MYC, a notion further supported by the strongly increased survival of TT-A1-derived B cells cultured *ex vivo* (Supplementary Figure 3b).

## Discussion

Using a constitutively active model of *in vivo* RNAi that allows simultaneous targeting of all A1 paralogues expressed in mice leading to ~50% reduction in protein expression, thereby circumventing possible adaptation processes associated with complete loss of gene function, we define anti-apoptotic Bcl2a1/A1 as a MYC target and facilitator of B cell transformation. In contrast, A1 appears dispensable for BCR-ABL or v-Abl-driven transformation and pre-B-ALL development, although aberrant kinase signaling, similar to MYC overexpression, led to an increase in A1 protein expression. On the basis of this, one may hypothesize that deregulation of ABL kinase that activates multiple signaling pathways enhancing proliferation and cell survival, including JAK/STAT and PI3K signaling next to MYC activation,^17, 23^ can engage multiple Bcl-2 prosurvival proteins, and that among those A1 exerts only a redundant role. Interestingly, however, the combined inhibition of the v-Abl kinase by imatinib and that of Bcl-2 proteins not under direct control of kinase signaling using ABT-737 enhances cell death in A1 knockdown cells. This assigns an unnoted survival role to A1 in Abl-transformed early B cell progenitors that is independent of kinase signaling and that may hence be exploited therapeutically by combinatorial drug targeting.

Our data are also in line with the finding that survival of pro- vs pre-B cells is differentially regulated during lymphocyte development where these cells seem to rely on *Mcl-1* and *Bcl-x*, respectively (reviewed in Sochalska *et al.*^24^). Evidently, these dependencies seem to change in response to oncogenic stress and may assign cell type dependent roles to A1 or other BCL-2 prosurvival homologues that cannot be anticipated from their expression pattern observed during normal hematopoiesis. This phenomenon is best exemplified by the fact that high levels of BCL-2 or BCL-X have been reported to be selected for in MYC-driven lymphomas,^25^ but deletion of *Bcl-2* had no impact on tumorigenesis, while loss of *Bcl-x* caused only a moderate delay in tumor latency.^4, 5^ In contrast, deletion of *Mcl-1* that was never reported as overexpressed in murine MYC-driven B cell tumors effectively precluded tumor formation and was actively selected against in mice that eventually developed disease.^22^ Similar findings were made in BCR-ABL-driven pre-B-ALL where loss of *Bcl-x* had no effect on tumorigenesis, whereas loss of *Mcl-1* prevented disease.^22^ Together, this suggests that MCL-1 is the key prosurvival BCL-2 protein for immature pro- and pre-B cells during oncogenic transformation and that one or the other BCL-2 family member may have auxiliary function, depending on cell type, differentiation stage or oncogenic driver. This is best seen for *Bcl-x* in the context of MYC overexpression. Based on our findings, A1 also appears to qualify as auxiliary factor in MYC-driven transformation. This notion is also underlined by the fact that the protumorigenic potential of exogenous A1 has been demonstrated before in MYC-driven B cell and Lck-driven T cell transformation.^26, 27^

Deregulation of BCL-2 family member expression is well known to associate with tumor progression and drug resistance in blood cancer and oncogenic kinases such as BCR-ABL. BCL-2 family members appear to repress apoptosis in part by inhibiting the effects of proapoptotic BH3-only proteins, such as BIM or BCL-2-associated death promoter.^18^ Based on our observation that v-Abl-transgenic tumor cells expressing the miR-shRNA targeting A1 were found more sensitive to imatinib, we conclude that v-Abl-kinase signaling promotes survival at least in part by increasing the ratio between A1 and BIM expression levels. Reducing this ratio can facilitate BIM-dependent cell death, as documented before in mature B cells exposed to SYK inhibitor that blocks BCR-dependent A1 expression.^14^ Along these lines, recent evidence uncovered that high expression of A1/BFL-1 mediates drug resistance to BRAF inhibitors that have also been shown to act in part by stabilizing BIM levels in human melanoma.^11^ Hence, the ratio between A1 and BIM may be decisive how well malignant cells can respond to therapy.

Similarly, MYC overexpression uncouples A1 expression from BCR signaling. Lowering A1 levels increased cell death of MYC-transgenic B cells that can be counteracted by IgM stimulation, shown to induce A1 protein (Figure 2), or by re-expression of RNAi resistant BFL1 (Figure 6). Similar to *Bim* that has been identified as a *bona fide* MYC target^28^ and the BCL2A1 promoter has been confirmed to among a large set of MYC binding loci mapped in human B cells.^29^ We thus hypothesize that co-regulation of A1 by MYC helps to antagonize the proapoptotic potential of BIM in the context of deregulated MYC expression, thereby facilitating transformation. As such, A1 may also be a limiting factor antagonizing cell death induced by extensive BCR cross-linking that appears to engage different BH3-only proteins, including BIM, in established Eμ-MYC-tumors.^30^ Most strikingly, this study shows also that resistance to apoptosis caused by loss of *Bim* can be ameliorated by inhibition of SYK that acts in part by reducing A1 expression.^14^

In light of the finding that the application of BH3-mimetics induces compensatory A1 overexpression in human CLL and NHL-model cell lines, further studies into the role of A1/BFL-1 as a survival and drug-resistance factor in human blood cancer are warranted. Of note, BFL1 is induced by epstein–barr virus infection in an LMP1-dependent manner, a situation frequently found in Burkitt lymphoma patients.^31^ It is currently unclear, however, whether A1 is needed for tumor maintenance. It is safe to predict that the selective inhibition of A1 has the potential to enhance effects/synergize with state-of-the-art anticancer therapy even when not active as single agent in the A1/BFL-1-dependent cancer cell type.

## Materials and methods

### Transgenic mice

Animal experiments were performed in accordance with Austrian legislation (BMWF: 66-011/0006-II/3b/2014). The generation and genotyping of VV-A1, VV-FF and Eμ-MYC mice have been described.^16, 20^ Vav-BFL1 mice were generated by pronucleus injection using the *VavP* vector^32^ encoding *BFL-1* cDNA. The generation of these mice will be described in detail elsewhere. All mice were maintained on a C57BL/6 genetic background. Disease assessment: signs of leukemia/lymphoma were monitored for by regular palpation three times a week, monitoring of body weight, short breath and/or scruffy fur as well as social behavior. Inclusion criteria for sacrifice: detection of enlarged lymph nodes or spleen by palpation, weight loss of >15% and short breath.

### Cell culture, virus production and reagents

All cells were cultured at 37 °C in a humidified atmosphere containing 5% CO_2_. v-Abl transduced pre-B cell lines and BaF3 cells (ATCC, Wesel, Germany) were grown in the RPMI-1640 medium (Sigma-Aldrich, Dorset, UK) supplemented with 10% FCS (Sigma-Aldrich, F7524), 250 μM L-glutamine (PAA laboratories, Pasching, Austria, M11-004), 100 U/ml penicillin, 100 μg/ml streptomycin (PAA laboratories, P11-010) and 50 μM 2-mercaptoethanol (Applicam, Darmstadt, Germany). Cell lines tested negative for mycoplasma contamination by PCR (Lonza, Basel, Switzerland). Primary hematopoietic cells were cultured in RPMI-1640 medium supplemented with 10% FCS, 250 μM L-glutamine, 50 μM 2-mercaptoethanol, 1 mM sodium pyruvate (Gibco, Waltham, MA, USA), 100 μM nonessential amino acids (Gibco) and antibiotics. To generate viral supernatants encoding for v-Abl, A010 cells were plated in 100 mm dishes, precoated with gelatine (1% in PBS) and grown to confluency. Supernatant was harvested every 8 h for 40 h, pooled and filtered through a 0.45 m filter prior to injection. For the induction of pre-B-ALL, newborn mice were injected subcutaneously with 100 μl of v-Abl-endcoding retroviral supernatant as described.^17^

### Antibodies, flow cytometric analysis and cell sorting

The monoclonal antibodies used were purchased from eBioscience (San Diego, CA, USA) BioLegend (London, UK) or BD (San Diego, CA, USA). Flow cytometric analysis or cell sorting was performed on an LSR-Fortessa or a FACS-Aria-III, respectively (both BD). Antibodies used for flow cytometry: T-cells: 53-6.7, anti-CD8 (25-0081-82, eBioscience); RM 4-5, anti-CD4 (45-0042-82, eBioscience); 3C7, anti-CD25 (12-0251-83, eBioscience); B-cells: RA3-6B2, anti-B220 (103224, Biolegend); 1B11, anti-CD43 (121204, Biolegend); 2B8, anti–c-Kit (105813, Biolegend); II/41, anti-IgM (406509, Biolegend); 11/26C, anti-IgD (12-5993-83, eBioscience); 6D5, anti-CD19 (115533, Biolegend); myeloid cells: RB6-8C5, anti–Gr-1 (108404, Biolegend); S7, MI/70, anti–Mac-1 (17-0112-83, eBioscience). Biotinylated antibodies were detected using streptavidin-RPE (DAKO, Glostrup, Denmark) or streptavidin coupled to PE-Cy7, APC, APC-Cy7 or PerCP5.5 (BioLegend or eBioscience).

### Immunoblotting

Cell lysates were prepared in CHAPS-containing lysis buffer and analyzed by immunoblotting as described.^16^ For detection of proteins by chemoluminescence (Advansta, Menlo Park, CA, USA, K-12049-D50) a rat anti-mA1 mAb, clone 6D6-1-1,^33^ rat anti-Bim, clone 3C5 (Enzo, Lausen, Switzerland), rabbit anti-Mcl1 (Rockland, Hamburg, Germany, 600-401-394) and a rabbit anti-GAPDH mAb (Cell Signaling, Beverly, MA, USA, 2118, 1:5000) were used. An anti-BFL-1 polyclonal rabbit serum was kindly provided by J Borst (NKI). Goat anti-rabbit Ig/HRP (Dako, P0448) or rabbit anti rat-IgG heavy chain-HRP (Cell Signaling, 7077) were used as secondary reagents.

### Survival assays and chemical compounds

Splenocytes were stimulated for 24 h with 100 U/ml of mIL-2, 10 ng/ml mIL-4, 10 ng/ml mIL-5 (all PeproTech, Rocky Hill, NJ, USA) and 1 μg/ml goat anti-mouse IgM F(ab′)_2_ fragments (Dianova, Hamburg, Germany), 1 μg/ml hamster anti-mouse CD40 mAb (BioLegend or BD) or 20 μg/ml or lipopolysaccharide (Sigma-Aldrich, L2880). Prior flow cytometric analysis, cells were stained with B220-PE and CD19-APC antibodies. The percentage of viable cells was determined using Annexin-V-Pacific Blue (eBioscience, 88-8006-74). Five cell lines derived from WT and five cell lines derived from diseased VV-A1 mice infected with v-Abl oncogene were treated with Imatinib (Selleckchem) or ABT-737 (Selleckchem), alone or in combination. SYK inhibitor R406 was purchased from Selleckchem. The MYC-selective inhibitors, 10058-F4 and 10074-G5, were kindly provided by Dr Markus Hartl, LFU Innsbruck.

### Statistical analysis

Statistical analysis was performed using ANOVA followed by Bonferoni *post hoc* test, applying the PRISM Graphpad software. *P* values <0.05 were considered to indicate statistically significant differences. For statistical analysis, mean values with s.e.m. are presented in graphs that were derived from several independent repeats of experiments. Bartlett's test for equal variances did not reveal any variances that were significantly different. Normal distribution was assessed (for data sets *n*⩾5) by the Kolmogorov–Smirnov test. For survival analysis of tumor mice, the log rank (Mantel–Cox) test was used.

## References

[bib1] Anderson MA, Huang D, Roberts A. Targeting BCL2 for the treatment of lymphoid malignancies. Semin Hematol 2014; 51: 219–227. 25048785 10.1053/j.seminhematol.2014.05.008

[bib2] Delbridge AR, Strasser A. The BCL-2 protein family, BH3-mimetics and cancer therapy. Cell Death Differ 2015; 22: 1071–1080. 25952548 10.1038/cdd.2015.50 PMC4572872

[bib3] Deng J, Carlson N, Takeyama K, Dal Cin P, Shipp M, Letai A. BH3 profiling identifies three distinct classes of apoptotic blocks to predict response to ABT-737 and conventional chemotherapeutic agents. Cancer Cell 2007; 12: 171–185. 17692808 10.1016/j.ccr.2007.07.001

[bib4] Kelly PN, Grabow S, Delbridge AR, Strasser A, Adams JM. Endogenous Bcl-xL is essential for Myc-driven lymphomagenesis in mice. Blood 2011; 118: 6380–6386. 21998213 10.1182/blood-2011-07-367672 PMC3236120

[bib5] Kelly PN, Puthalakath H, Adams JM, Strasser A. Endogenous bcl-2 is not required for the development of Emu-myc-induced B-cell lymphoma. Blood 2007; 109: 4907–4913. 17317859 10.1182/blood-2006-10-051847 PMC1885522

[bib6] Ottina E, Tischner D, Herold MJ, Villunger A. A1/Bfl-1 in leukocyte development and cell death. Exp Cell Res 2012; 318: 1291–1303. 22342458 10.1016/j.yexcr.2012.01.021 PMC3405526

[bib7] Vogler M. BCL2A1: the underdog in the BCL2 family. Cell Death Differ 2011; 19: 67–74. 22075983 10.1038/cdd.2011.158 PMC3252829

[bib8] Morales AA, Olsson A, Celsing F, Osterborg A, Jondal M, Osorio LM. High expression of bfl-1 contributes to the apoptosis resistant phenotype in B-cell chronic lymphocytic leukemia. Int J Cancer 2005; 113: 730–737. 15499630 10.1002/ijc.20614

[bib9] Vogler M, Butterworth M, Majid A, Walewska RJ, Sun XM, Dyer MJ et al. Concurrent up-regulation of BCL-XL and BCL2A1 induces approximately 1000-fold resistance to ABT-737 in chronic lymphocytic leukemia. Blood 2009; 113: 4403–4413. 19008458 10.1182/blood-2008-08-173310

[bib10] Yecies D, Carlson NE, Deng J, Letai A. Acquired resistance to ABT-737 in lymphoma cells that up-regulate MCL-1 and BFL-1. Blood 2011; 115: 3304–3313. 10.1182/blood-2009-07-233304 PMC2858493 20197552

[bib11] Haq R, Yokoyama S, Hawryluk EB, Jonsson GB, Frederick DT, McHenry K et al. BCL2A1 is a lineage-specific antiapoptotic melanoma oncogene that confers resistance to BRAF inhibition. Proc Natl Acad Sci USA 2013; 110: 4321–4326. 23447565 10.1073/pnas.1205575110 PMC3600451

[bib12] Hatakeyama S, Hamasaki A, Negishi I, Loh DY, Sendo F, Nakayama K. Multiple gene duplication and expression of mouse bcl-2-related genes, A1. Int Immunol 1998; 10: 631–637. 9645611 10.1093/intimm/10.5.631

[bib13] Vier J, Groth M, Sochalska M, Kirschnek S. The anti-apoptotic Bcl-2 family protein A1/Bfl-1 regulates neutrophil survival and homeostasis and is controlled via PI3K and JAK/STAT signaling. Cell Death Dis 2016; 7: e2103. 26890142 10.1038/cddis.2016.23 PMC5399193

[bib14] Sochalska M, Ottina E, Tuzlak S, Herzog S, Herold M, Villunger A. Conditional knockdown of BCL2A1 reveals rate-limiting roles in BCR-dependent B-cell survival. Cell Death Differ 2016; 23: 628–639. 26450454 10.1038/cdd.2015.130 PMC4986635

[bib15] Ottina E, Lyberg K, Sochalska M, Villunger A, Nilsson G. Knockdown of the anti-apoptotic Bcl-2-family member A1/Bfl-1 protects mice from anaphylaxis. J Immunol 2015; 194: 1316–1322. 25548219 10.4049/jimmunol.1400637 PMC4298126

[bib16] Ottina E, Grespi F, Tischner D, Soratroi C, Geley S, Ploner A et al. Targeting antiapoptotic A1/Bfl-1 by *in vivo* RNAi reveals multiple roles in leukocyte development in mice. Blood 2012; 119: 6032–6042. 22581448 10.1182/blood-2011-12-399089 PMC3418769

[bib17] Hoelbl A, Schuster C, Kovacic B, Zhu B, Wickre M, Hoelzl MA et al. Stat5 is indispensable for the maintenance of bcr/abl-positive leukaemia. EMBO Mol Med 2011; 2: 98–110. 10.1002/emmm.201000062 PMC2906698 20201032

[bib18] Kuroda J, Puthalakath H, Cragg MS, Kelly PN, Bouillet P, Huang DC et al. Bim and Bad mediate imatinib-induced killing of Bcr/Abl+ leukemic cells, and resistance due to their loss is overcome by a BH3 mimetic. Proc Natl Acad Sci USA 2006; 103: 14907–14912. 16997913 10.1073/pnas.0606176103 PMC1595449

[bib19] Oltersdorf T, Elmore SW, Shoemaker AR, Armstrong RC, Augeri DJ, Belli BA et al. An inhibitor of Bcl-2 family proteins induces regression of solid tumours. Nature 2005; 435: 677–681. 15902208 10.1038/nature03579

[bib20] Adams JM, Harris AW, Pinkert CA, Corcoran LM, Alexander WS, Cory S et al. The c-*myc* oncogene driven by immunoglobulin enhancers induces lymphoid malignancy in transgenic mice. Nature 1985; 318: 533–538. 3906410 10.1038/318533a0

[bib21] Glaser SP, Lee EF, Trounson E, Bouillet P, Wei A, Fairlie WD et al. Anti-apoptotic Mcl-1 is essential for the development and sustained growth of acute myeloid leukemia. Genes Dev 2012; 26: 120–125. 22279045 10.1101/gad.182980.111 PMC3273836

[bib22] Kelly GL, Grabow S, Glaser SP, Fitzsimmons L, Aubrey BJ, Okamoto T et al. Targeting of MCL-1 kills MYC-driven mouse and human lymphomas even when they bear mutations in p53. Genes Dev 2014; 28: 58–70. 24395247 10.1101/gad.232009.113 PMC3894413

[bib23] Warsch W, Walz C, Sexl V. JAK of all trades: JAK2-STAT5 as novel therapeutic targets in BCR-ABL1+ chronic myeloid leukemia. Blood 2013; 122: 2167–2175. 23926299 10.1182/blood-2013-02-485573

[bib24] Sochalska M, Tuzlak S, Egle A, Villunger A. Lessons from gain- and loss-of-function models of pro-survival Bcl2 family proteins: implications for targeted therapy. FEBS J 2015; 282: 834–849. 25559680 10.1111/febs.13188 PMC4562365

[bib25] Eischen CM, Woo D, Roussel MF, Cleveland JL. Apoptosis triggered by *myc*-induced suppression of Bcl-X_L_ or Bcl-2 Is bypassed during lymphomagenesis. Mol Cell Biol 2001; 21: 5063–5070. 11438662 10.1128/MCB.21.15.5063-5070.2001 PMC87232

[bib26] Fan G, Simmons MJ, Ge S, Dutta-Simmons J, Kucharczak J, Ron Y et al. Defective ubiquitin-mediated degradation of antiapoptotic Bfl-1 predisposes to lymphoma. Blood 2011; 115: 3559–3569. 10.1182/blood-2009-08-236760 PMC2867266 20185581

[bib27] Beverly LJ, Varmus HE. MYC-induced myeloid leukemogenesis is accelerated by all six members of the antiapoptotic BCL family. Oncogene 2009; 28: 1274–1279. 19137012 10.1038/onc.2008.466 PMC2743088

[bib28] Muthalagu N, Junttila MR, Wiese KE, Wolf E, Morton J, Bauer B et al. BIM is the primary mediator of MYC-induced apoptosis in multiple solid tissues. Cell Reports 2014; 8: 1347–1353. 25176652 10.1016/j.celrep.2014.07.057 PMC4231288

[bib29] Zeller KI, Zhao X, Lee CW, Chiu KP, Yao F, Yustein JT et al. Global mapping of c-Myc binding sites and target gene networks in human B cells. Proc Natl Acad Sci USA 2006; 103: 17834–17839. 17093053 10.1073/pnas.0604129103 PMC1635161

[bib30] Carter MJ, Cox KL, Blakemore SJ, Bogdanov YD, Happo L, Scott CL et al. BCR-signaling-induced cell death demonstrates dependency on multiple BH3-only proteins in a murine model of B-cell lymphoma. Cell Death Differ 2016; 23: 303–312. 26184912 10.1038/cdd.2015.97 PMC4716310

[bib31] Pegman PM, Smith SM, D'Souza BN, Loughran ST, Maier S, Kempkes B et al. Epstein-Barr virus nuclear antigen 2 trans-activates the cellular antiapoptotic bfl-1 gene by a CBF1/RBPJ kappa-dependent pathway. J Virol 2006; 80: 8133–8144. 16873269 10.1128/JVI.00278-06 PMC1563820

[bib32] Ogilvy S, Metcalf D, Gibson L, Bath ML, Harris AW, Adams JM. Promoter elements of *vav* drive transgene expression *in vivo* throughout the hematopoietic compartment. Blood 1999; 94: 1855–1863. 10477714

[bib33] Lang MJ, Brennan MS, O'Reilly LA, Ottina E, Czabotar PE, Whitlock E et al. Characterisation of a novel A1-specific monoclonal antibody. Cell Death Dis 2014; 5: e1553. 25476901 10.1038/cddis.2014.519 PMC4649835

