# Supplementary material for: MYC selects against reduced BCL2A1/A1 protein expression during B cell lymphomagenesis
Source: Oncogene. 2016 Oct 3;36(15):2066–73. doi: 10.1038/onc.2016.362 (PMC5395700; doi:10.1038/onc.2016.362)
Supplement: Supplementary Information [file onc2016362x1.pdf]

Suppl. Figure legend

Fig. S1. (a) Quantification of Venus expression in major organs of VV-A1 and VV-FF mice in the absence of exogenous MYC expression. (*$P \leq .05$, **$P \leq .01$, unpaired t-test Bars represent means ± SEM, VV-FF n=3, VV-A1 n=4, biological replicates, two independent experiments). **(b)** Quantification of different B cell subsets in the indicated lymphatic organs based on Venus expression. Single cell suspensions were stained with antibodies recognizing B220, IgM or IgD. $B220^{+}IgD^{-}IgM^{-}$ represent pro/pre-B cells, $B220^{+}IgD^{-}IgM^{+}$ immature B cells and $B220^{+}IgD^{+}IgM^{-}$ recirculating B cells. Bars represent means ± SEM (WT n=3, VV-FF n=3, VV-A1 n=4, biological replicates, two independent experiments). Abbreviations: BM = bone marrow, LN = lymph node. No significant differences were noted.

Fig. S2. (a) Representative dot plots of Venus expression assessed by flow cytometry in different hematopoietic organs of premalignant DT-FF and DT-A1 mice vs. Eμ-MYC single transgenic control mice.

Fig. S3. (a) Evaluation of BFL-1 transgene expression in hematopoetic organs in F1 offspring from three different *Vav-BFL1* transgenic founders. Line L3 was used for intercrossing with DT-A1 mice to generate triple transgenic mice (TT-A1) and data shown in Figure 6. (**b**) Total spleen cells from mice of the indicated genotypes were incubated in the presence or absence of the indicated mitogens for 24h. The percentages of viable (Annexin V^{-}) $CD19^{+}B220^{+}$ B cells in the $Venus^{-}$ or $Venus^{+}$ fractions derived from DT-A1 or triple transgeni (TT-A1) mice or the total cell fraction of MYC or DT-FF mice (>90%

Venus$^+$ cells) was assessed by flow cytometry. Bars represent means of two independent experiments performed in duplicates ± SD.

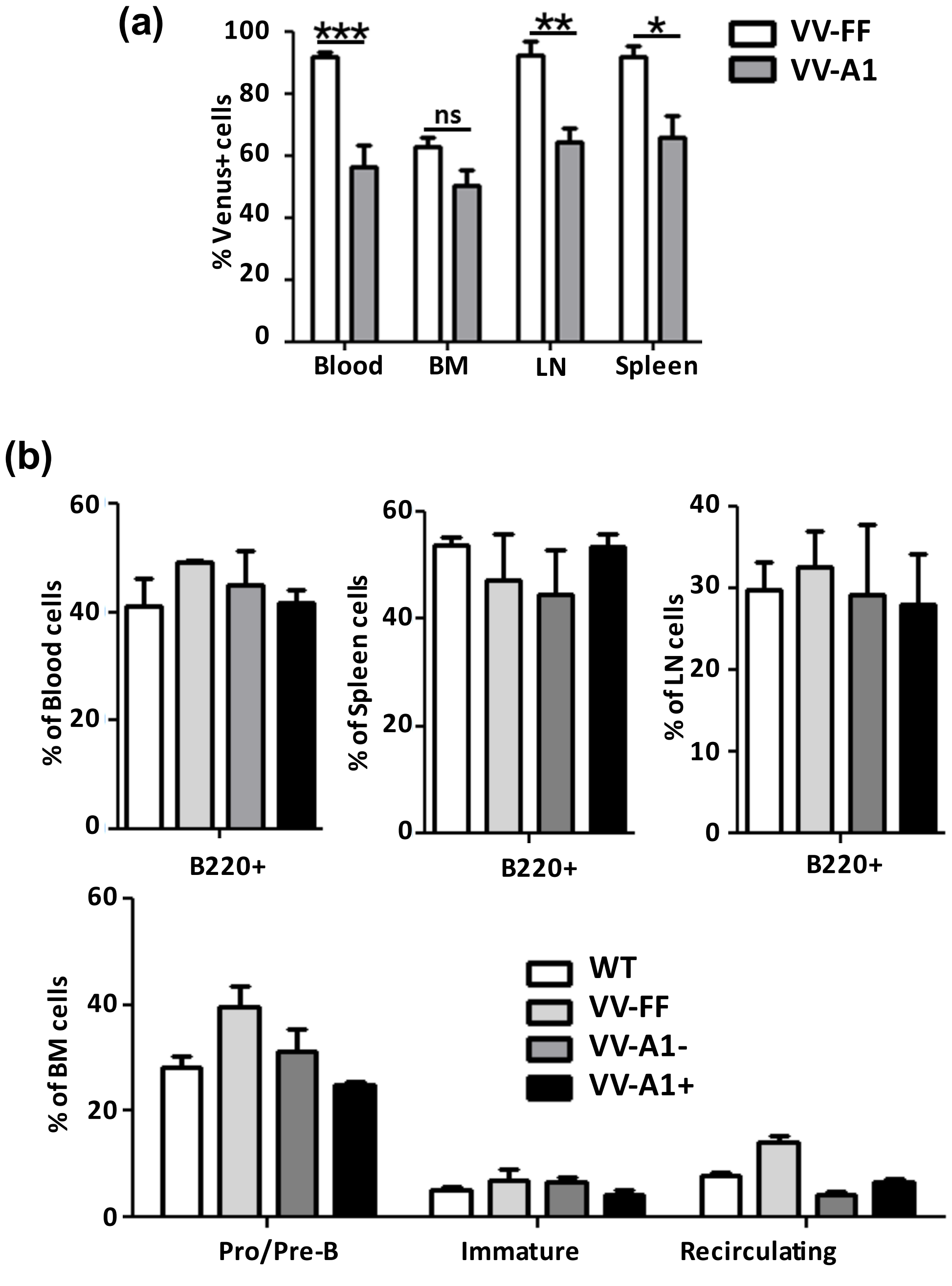
(a)
100
80
60
40
20
0
% Venus+ cells
***
ns
**
*
Blood
BM
LN
Spleen
VV-FF
VV-A1
(b)
60
40
20
0
% of Blood cells
B220+
60
40
20
0
% of Spleen cells
B220+
40
30
20
10
0
% of LN cells
B220+
60
40
20
0
% of BM cells
WT
VV-FF
VV-A1-
VV-A1+
Pro/Pre-B
Immature
Recirculating

Sochalska et al_Suppl. Fig. 2

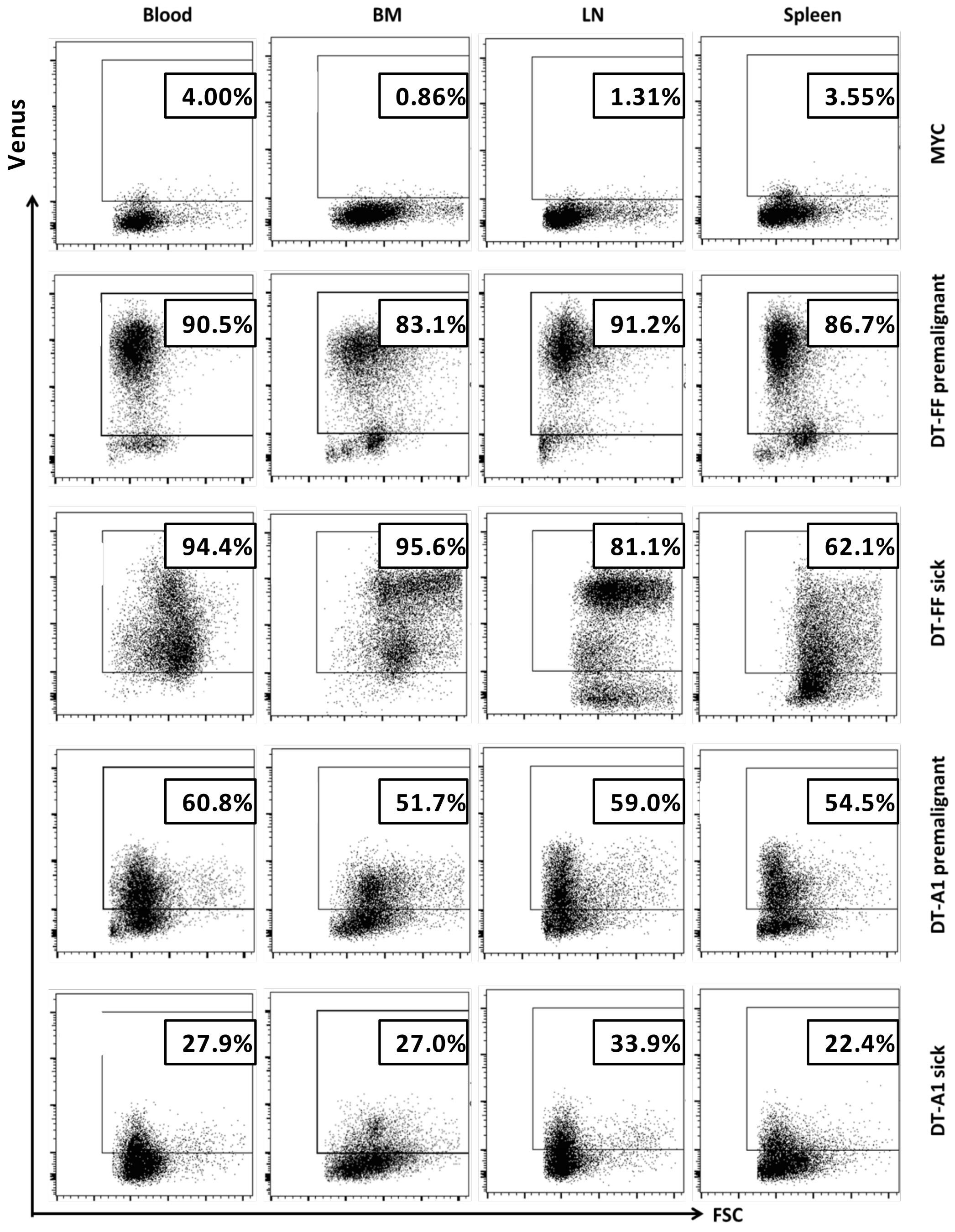

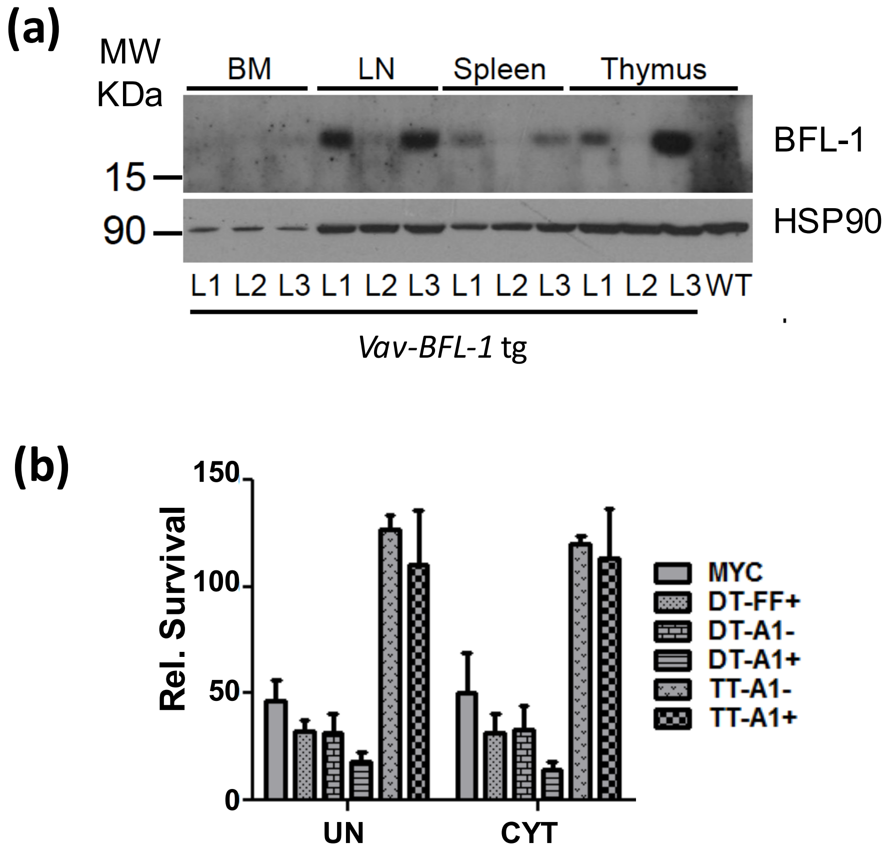
(a)
MW
KDa
BM
LN
Spleen
Thymus
BFL-1
15
90
HSP90
L1 L2 L3 L1 L2 L3 L1 L2 L3 L1 L2 L3 WT
Vav-BFL-1 tg
(b)
150
100
50
0
Rel. Survival
UN
CYT
MYC
DT-FF+
DT-A1-
DT-A1+
TT-A1-
TT-A1+
